# The Influence of Field Teaching Practice on Pre-service Teachers’ Professional Identity: A Mixed Methods Study

**DOI:** 10.3389/fpsyg.2017.01264

**Published:** 2017-07-24

**Authors:** Hongyu Zhao, Xiaohui Zhang

**Affiliations:** ^1^Faculty of Psychology, Beijing Normal University Beijing, China; ^2^Haidian Institute of Education Sciences Beijing, China

**Keywords:** field teaching practice, internship, pre-service teacher, professional identity, mentor support, professional development

## Abstract

The current study used mixed methods to research pre-service teachers’ professional identity. Ninety-eight pre-service teachers were investigated and twelve teachers were interviewed in China. The results were as follows: (1) The results of quantitative data showed that compared with before the field teaching practice, pre-service teachers’ professional identity increased after the field teaching practice—specifically, intrinsic value identity increased, and extrinsic value identity did not significantly change; (2) The results of qualitative data validated and elaborated the results of quantitative data in more detail with regard to changes in professional identity. Specifically, compared with before the field teaching practice, intrinsic value identity including work content, work pattern, etc., increased and extrinsic value identity including work environment, income, and social status, etc., did not significantly change after experiencing teaching practice; (3) The results of qualitative data also showed that mentor support at field school promoted the development of pre-service teachers’ professional identity. Moreover, the development of pre-service teachers’ professional identity during field teaching practice further promoted their professional commitment; that is, it promoted their emotional evaluation and belief in the teaching profession. The study discussed these results and proposed solutions and suggestions for future studies.

## Introduction

### Teachers’ Professional Identity

Teachers’ professional identity is an important research field. It is a core element of teachers’ professional lives, and also a “resource that people use to explain, justify, and make sense of themselves in relation to others, and to the world at large” (MacLure, 1993, p. 311). Teachers’ professional identity has a widespread effect on a teachers’ teaching, professional development, and staying in the teaching profession, etc., and influences individual teaching effects by affecting their concrete behaviors in the process of teaching (Korthagen, 2004). Teachers’ short- and long-term decisions about curriculum design, pedagogy, assessment, and student learning are limited by their understanding of their teacher identity (Mockler, 2011). Sammons et al. (2007) conducted a study with a large-scale, longitudinal research in England. The results found a relationship between aspects of teachers’ professional identity and pupils’ attainments in English and mathematics. Zhang et al. (2016) conducted a study with pre-service teachers in the stage of teacher education. The results showed pre-service teachers’ professional identity influenced program performance by affecting their task value belief and extrinsic learning motivation. Moore and Hofman (1988) found that teachers with lower professional identity easily perceived lower work satisfaction and higher work stress, and teachers with higher professional identity were more likely to overcome the dissatisfaction with harsh working conditions. Moore and Hofman (1988) and Gaziel (1995) found professional identity related to intentions to leave the teaching profession. Furthermore, the influence of professional identity on the development of teaching practice received more attention in the research. Bullough and Gitlin (2001) also emphasized that the crucial role of professional identity in the stage of the teaching practice should be paid attention to.

However, scholars have different definitions for teachers’ professional identity. Some researchers defined teachers’ professional identity from the perspective of self or self-concept. For example, Kelchtermans (2000) argued that the teaching profession is highly self-involved and that teachers’ professional identity is a concept of the teacher as a teacher. Volkmann and Anderson (1998) deemed that the teaching profession requires a complex and dynamic equilibrium between personal self-image and teacher roles. Akkerman and Meijer (2011) proposed that the formation of teachers’ professional identity is a process of narrating and relating multiple I-positions and it is formed in the course of self-participation and self-engagement and in the course of trying to maintain continuity and consistency. Actually, the self is formed in the process of complex and meaningful social interaction; without environmental factors or professional backgrounds, there is no self or professional identity.

In view of the close relationship between identity and profession, Tickle (2000) proposed that professional identity is embodied through professional characteristics. Many researchers defined teachers’ professional identity based on Tickle’s perspective. For example, Nixon (1996) believed that teachers’ professional identity is something that characterizes an occupational group with specific working conditions. Gaziel (1995) argued that teachers’ professional identity is similar to a list of items that represents aspects of the profession. Young and Graham (1998) defined teachers’ professional identity as the characteristics of an ideal teacher. Schepens et al. (2009) tested the relationship between professional identity and educational situation through professional characteristics. Based on professional knowledge and skills teacher obtained, Beijaard et al. (2000) divided teachers’ professional identity into three aspects—subject matter, didactic, and pedagogical expertise.

Indeed, professional identity is regarded as an attitude from a psychological point of view. Attitude is an evaluative statement about things, persons, and events; the evaluative statement, approved or rejected, reflects a person’s emotional object (Robbins and Judge, 2007). Accordingly, professional identity is based on cognitive and emotional elements. However, as an attitude, these elements are not discrete but a combination of cognition and emotion; the two elements are inseparable and are displayed in judgments of value. Therefore, the present study defines professional identity as an attitude; professional identity involves teachers making a judgment or assessment of the importance and value of the teaching profession’s different characteristics. Meanwhile, many scholars considered teachers’ profession is multifaceted; for example, Moore and Hofman (1988) believed that teachers’ professional identity includes centrality, valence, consonance, and self-presentation. Kelchtermans (2009) deemed that teachers’ professional identity consists of self-image, self-esteem, job motivation, task perception, and future perspectives. Hong (2010) considered that teachers’ professional identity is composed of value, self-efficacy, commitment, emotions, knowledge and beliefs, micropolitics, and so on. As stated above, the value of the teaching profession is a core element of professional identity; meanwhile, professional identity also includes other elements that vary with different teacher groups and professional development stages.

Compared with in-service teachers, pre-service teachers lack real experiences of the teaching profession, and their cognition and evaluation of the teaching profession are more based on teachers as students. For this reason, some scholars believe that pre-service teachers have not yet formed an essential professional identity but just formed a student identity (Beauchamp and Thomas, 2006; Flores and Day, 2006; Levin and He, 2008). Hong (2010) found that pre-service teachers’ attitudes to their profession were vague, while in-service teachers’ attitudes to their profession were specific and realistic, including in the areas of classroom control, knowledge teaching, and relationships with parents, colleagues, and managers. Therefore, under the influence of the career development stage, the structure of pre-service teachers’ professional identity is relatively simple; it is likely to mainly focus on the value of the teaching profession.

Zhang (2016) proposed a pre-service teachers’ professional identity model composed of intrinsic value identity and extrinsic value identity. Intrinsic value identity is mainly related to individuals’ subjective feelings regarding the inherent feature of the teaching profession, such as work contents and work characteristics. Extrinsic value identity mainly focuses on cognitions about the external feature of the teaching profession, such as work environment, social status, and income. The present study will investigate and analyze pre-service teachers’ professional identity from these two dimensions.

### The Development of Pre-service Teachers’ Professional Identity during Field Teaching Practice

In view of the characteristics of pre-service teachers’ professional identity, some scholars proposed that pre-service teachers would experience an intricate transition of professional identity in the stage of teacher education; that is, their professional identity would constantly experience negotiation, construction, and acceptance. However, fundamental change was less likely to occur (Korthagen, 2004). The transition of identity was a difficult and slow process, and even if pre-service teachers entered the teacher education stage, their belief, and cognition were still stubborn, and they tended to use the knowledge and information teacher education provided to confirm rather than confront and adjust their original beliefs and cognition. In general, when pre-service teachers engaged in teaching and internship, they did not have sufficient knowledge about students and the classroom, and they brought unrealistic views and optimistic attitudes to the classroom and treated classroom practice from an oversimplified viewpoint (Kagan, 1992).

However, this did not mean that pre-service teachers’ professional identity could not change during the whole teacher education stage. Under the influence of individual internal and external factors, and subjective and objective factors, pre-service teachers’ professional identity could not change in structure, but the dimensions of professional identity possibly changed on some level. Some studies found that pre-service teachers tended to overestimate their professional commitment and professional efficacy before the internship (Volkmann and Anderson, 1998; Kelchtermans and Ballet, 2002). The main reason for this was that pre-service teachers often underestimated the complexity of the teaching profession before they entered into the internship. The aim of the internship was to pull them back to reality from theoretical learning, which led to a decrease in professional commitment and professional efficacy. Hong (2010) found that the scores of students who experienced internship were lower than those of students who did not, on the emotion dimension of professional identity, which was possibly related to emotional exhaustion of students during the internship. Certainly, the changes in pre-service teachers’ professional identity before and after the internship were related to different definitions of professional identity and also related to some factors in the teacher education stage. For example, Johnson and Ridley (2004) found that providing support for pre-service teachers, including providing guidance in the initial teaching jobs for novice teacher, integrating school culture, communicating class plan with expert teacher, etc., decreased the difficulty of transition from student to teacher. A study on novice teachers showed that the support and positive feedback from supervisor, assistants, and parents affected the success and well-being of novice teachers (Avalos and Aylwin, 2007; Oplatka and Eizenberg, 2007).

Moreover, there were many studies on teachers’ professional identity, but the main method used was qualitative research, such as teachers’ reinvention (e.g., Mitchell and Weber, 1999), creative narratives, discourses of teaching lives (e.g., Sfard and Prusak, 2005; Alsup, 2006); the metaphors of a teacher’s role (e.g., Hunt, 2006; Leavy et al., 2007); and structured or semi-structured interviews, observations, written reflections, and self-recording (e.g., Palmér, 2015; Yuan and Lee, 2015; Izadinia, 2016). Limited quantitative research was mainly a cross-sectional study. For example, Hong (2010) conducted a cross-sectional and quantitative study with four groups, including pre-service teachers experiencing internship, pre-service teachers not experiencing internship, dropout teachers, and non-dropout teachers. Mahmoudi-Gahrouei et al. (2016) conducted a cross-sectional and quantitative study with three groups, including prospective teachers, new teachers, and experienced teachers. Therefore, longitudinal research on the changes in pre-service teachers’ professional identity before and after an internship is needed. Moreover, based on the quantitative study, the study further explores and elaborates on the changes, effects, and roles of pre-service teachers’ professional identity through a qualitative study.

### Summary

The development of teachers’ professional identity is a long-term process. This process starts from individual choices of teacher education (Walkington, 2005). Pre-service teachers experience the transition of professional identity in the process of situation transition from teacher education to internship, and further change of identity occurs in the whole process of teachers’ careers (Beauchamp and Thomas, 2009). Therefore, whether and how teaching practice promotes pre-service teachers’ professional identity is an important research topic (Beijaard et al., 2004; Korthagen, 2004).

In conclusion, as a course bridging theory and practice, field teaching practice is an important part of teacher education programs, and it plays a significant role in the formation and development of teachers’ professional identity. Therefore, a research mixing quantitative and qualitative approaches to investigate the changes of pre-service teachers’ professional identity during an internship and analysis of the reasons and factors behind the development is needed.

## Materials and Methods

This study employed a mixed-methods design that used a combination of qualitative and quantitative approaches. According to Creswell’s (2003) classification, the current study can be identified as a “the concurrent triangulation approach.” The mixed approach offsets the inherent weaknesses within one method with the strengths of the other (Creswell, 2009). Triangulation refers to “the combination of methodologies in the study of the same phenomenon” (Denzin, 1978), and this approach allows the researcher to improve the accuracy of conclusions by relying on data from more than one method (Rossman and Wilson, 1985). In this study, the quantitative survey and qualitative interview are concurrent, but greater weight is given to the qualitative approach. In this study, quantitative research including two time points was a longitudinal study, which was used to gain an overview of the development of pre-service teachers’ professional identity during their internship. Then, qualitative research was conducted to illustrate and elaborate the development in more detail, and to explore the factors influencing pre-service teachers’ professional identity and professional development in the future.

### Participants

Participants for the quantitative study were randomly sampled from different departments of a university in China. The curriculum and theoretical learning of teacher education mainly focused on Grades 1–3. Internship is conducted in Grade 4 in China. According to the regulation of the college, teaching practice is one of the requirements to obtain teacher certification. The contents of teaching practice mainly involve classroom teaching, class management, and other jobs. The internship continues for 16 weeks and includes 320 class hours. After finishing the internship, pre-service teachers obtain 10 credits according to standard requirements. Therefore, the first survey was conducted before the internship (Time 1), and the second survey at the end of the internship (Time 2).

Because most pre-service teachers were at the end of their internship and were looking for a job at the time of the second survey, some participants were lost because of lack of data in Time 2. Participants were 98 pre-service teachers who remained from the previous sample of 140 pre-service teachers (**Table 1**). There were 98 valid participants in the two tests. A comparison of the 42 lost participants to the remaining 98 participants showed that there were no group differences in gender [χ^2^(1) = 0.83, *p* = 0.36], student origin [χ^2^(2) = 1.49, *p* = 0.47], and teacher professional identity [*t*(1,138) = -0.79, *p* = 0.43]. Of the 98 participants, 82% were girls, 77% were science, 32% came from city, 35% came from town, and 33% came from the country.

**Table 1:** Participants’ demographic information.

Test	*N*	Gender		Source of students			Major	
		Male	Female	City	Town	Country	Arts	Science
The first test	140	30	110	51	42	47	43	97
The second test	98	18	80	31	34	33	22	76

Using the typical-case-sampling method, 12 (four males, eight females, average age = 21.78 years old) out of the 98 participants were selected for participation in the interview in the qualitative study. To ensure the representativeness of the sampling, the study considered participants’ gender, major, place of college admission, type of field school, type and subject of teaching, and so on. Their majors were different, with two students majoring in chemistry, two in literature, two in English, three in physics, two in educational technology, and one in special education. They conducted their field teaching practice at different middle schools located in different regions, such as Beijing, Hebei Province, Inner Mongolia, and Xinjiang Autonomous Regions. These middle schools varied in type from provincial or regional key schools to regular middle schools and schools for children with disabilities. The courses they taught varied from Chinese, math, English, chemistry, and physics to special education, according to their majors. The study was approved by the Ethics Committee of the Faculty of Psychology, Beijing Normal University, and all the work was carried out within the guidelines set by the committee. All subjects gave written informed consent in accordance with the Declaration of Helsinki.

Morse (1994) considered six participants as the smallest qualified number in phenomenological studies, and Kuzel (1992) argued that six to eight participants were acceptable in studies of homogeneous participants. The present study chose twelve participants for the research, and the sample size met the criterion mentioned. Additionally, according to the definition of “theoretical saturation” by Glaser and Strauss (1967) (theoretical saturation refers to a data size in which the researchers can no longer form a new category with additional data). Based on the sorting and coding of the interview data, we found that our domains and categories had reached theoretical saturation after the seventh participant’s data were analyzed. The data from the eighth to the twelfth participant could not form a new category code. This finding supported that twelve participants was an appropriate sampling number for this study.

### Data Collection and Analysis

#### Quantitative Data: Survey Questionnaire

The quantitative study used a survey questionnaire to investigate the changes of pre-service teachers’ professional identity. The participants were required to complete a questionnaire repeatedly before and after the internship.

To measure pre-service teachers’ professional identity, a validated scale was used in prior research with mainland Chinese samples (Zhang, 2016). The scale consisted of 10 items, for which students responded on a 6-point Likert self-report scale ranging from 1 (strongly disagree) to 6 (strongly agree). Two dimensions were contained in the scale: intrinsic value identity, which mainly focused on individual subjective value judgment of the inherent features of the teaching profession (seven items, e.g., “Teaching job is valuable” “Teaching job is attractive”); and extrinsic value identity, which mainly related to cognition of external features of professional identity (three items, e.g., “Teacher’s social status is high” “I think the work environment and condition for teacher are great”). The internal consistency reliabilities of the whole scale and the dimensions range from 0.79 to 0.87. The scale also has good validity. The results of confirmatory factor analysis showed that the two-factor model fit the data adequately: χ^2^ = 120.49, *df* = 26, RMSEA = 0.069, CFI = 0.98, TLI = 0.96, GFI = 0.97, NFI = 0.97. In this study, the reliability of the scale was acceptable. Cronbach’s alpha coefficient for the whole scale and the two dimensions in Time 1 and Time 2 ranged from 0.74 to 0.87.

#### Qualitative Data: Semi-structured Interview

The interviews were semi-structured and were administered by the chief researcher with assistance from other researchers. Participants were selected based on the representativeness of the sample and then contacted by the researcher. After the participants were invited to the lab, they were first asked to read and sign an informed consent form that described the purpose, safety, and privacy protection policy of the research and the recording notification. Each participant was interviewed once, the time of interview lasted 30 min, the interview was conducted one on one and recorded, on completion, the participants were thanked and given a gift.

The interviews involved structured questions and follow-up questions. Each participant was asked to answer the same questions, and specific questions or follow-up questions were added according to the initial answers. The interview protocol had three parts. First, the current study collected participants’ demographic information, including gender, age, internship time, place, school, subject, etc. Second, based on the definition of teachers’ professional identity for participants, the formal interview had two questions: (1) Compared with their professional identities before the internship, did participants’ professional identities change after the internship? What did these changes include? (2) Describe the causes of the changes, list the important people or events related to these causes, and answer additional further questions if necessary. Third, an open-ended interview was designed, and participants were asked to discuss the influence of these changes in professional identity on their future career development.

Audio recordings of the interviews were transcribed by the research team and then were checked carefully by non-team members to ensure accuracy. For data analysis, we adopted the consensual qualitative research (CQR) method (Miles and Huberman, 1994). The CQR method consists of domain coding, core idea coding, cross-case analysis, stability check, and audits. The key of the CQR method is a thorough team discussion of the transcribed data to reach an agreement on the conceptualization of the data. Our specific procedure was as follows: first, the interview data were grouped into several domains reflecting the main topics. Second, core ideas were extracted from each domain based on the interview contents and then examined in the context of each interview to ensure they indeed represented and covered related points of view. Third, categories and sub-categories were identified. Team members collected all of the core ideas from each domain for a cross-case analysis, found the common topics, clustered these topics to form categories and sub-categories, and then formed conclusions. In the CQR method, coding group members conducted the main analysis independently. All members needed to reach an agreement through consultations during the coding process. Finally, the coding results were submitted to external auditors. The external auditor re-examined the analysis and provided feedback to the coding group to refine the coding.

## Results

### The Changes in Professional Identity: Quantitative Findings

To investigate the changes in pre-service teachers’ professional identity before and after the internship, a paired-samples *t*-test was conducted. The results showed that there was significant difference in overall professional identity (*t*(97) = -2.26, 95% CI [-0.27, -0.02], *p* = 0.03), intrinsic value identity (*t*(97) = -4.12, 95% CI [-0.38, -0.13], *p* = 0.00), and extrinsic value identity (*t*(97) = -0.31, 95% CI [-0.22, 0.16], *p* = 0.76). **Table 2** showed mean, standard deviation, minimum and maximum, on each sub-dimension and overall construct. The results indicated that the overall professional identity increased, intrinsic value identity increased, and extrinsic value identity kept steady. Specifically, pre-service teachers with higher professional identity tended to think teaching is more valuable, more attractive, more interesting, and think communication with students is more meaningful. Meanwhile, extrinsic value identity, such as work environment and condition, social status, did not significant change. These results also implied that the inner tension of the two dimensions of teachers’ professional identity increased after the internship.

**Table 2:** Descriptive statistics of sub-dimensions and overall construct (*n* = 98).

Variable	Time	Min	Max	Mean	*SD*
Overall professional identity	Time 1	1.93	6.00	4.26	0.78
	Time 2	2.64	6.00	4.40	0.70
Intrinsic value identity	Time 1	2.57	6.00	4.58	0.74
	Time 2	2.86	6.00	4.84	0.69
Extrinsic value identity	Time 1	1.00	6.00	3.93	1.00
	Time 2	1.33	6.00	3.96	1.00

In summary, compared with the professional identity before the internship, the overall professional identity and intrinsic value identity increased, and extrinsic value identity kept steady after the internship. Why did intrinsic value identity and the overall professional identity increased after internship? Which factors influenced pre-service teachers’ professional identity during the field teaching practice? Did field teaching practice influence pre-service teachers’ professional development? To answer the above questions, a qualitative study was conducted.

### The Changes, Factors, and Roles of Identity: Qualitative Findings

The data analysis indicated that three domains—changes in identity, factors of identity and, roles of identity—could explain the changes in pre-service teachers’ professional identity before and after the internship and the influence of the internship on pre-service teachers’ professional identity and professional development. **Table 3** displayed domains, categories and sub-categories, representativeness of each category, and examples of the core ideas. According to Miles and Huberman (1994), each domain and category was identified by the frequency of usage. In the present study, we considered domain or category to be “general” if it was used in all 12 cases, “typical” if it was used in 8–11 cases, “variant” if used in 4–7 cases, and “insufficient of representativeness” if used in fewer than four cases.

**Table 3:** The results of the qualitative research: representativeness and examples of domain, category, and sub-category.

Domain, category, and sub-category	Number of times (*N* = 12)	Level ofrepresentativeness	Examples of core idea
**Identity changes**			
*Intrinsic value identity*			
Work contents	12	General	Before practice: I did not know much about teaching, and I thought that teachers only teach and manage class. After practice: I found that teachers also need to do the work assigned by the school and at the same time still pay attention to communication with students.
Work characteristic	11	Typical	Before practice: Teaching is boring, especially when teachers have to devote their whole life to only a few textbooks. After practice: Teaching can be creative, not just a simple repetition.
*Extrinsic value identity*			
Work environment	4	Variant	Before practice: The work environment is good, and social interactions are simple and pure and not complicated. After practice: The view is the same as before the practice.
Income	9	Typical	Before practice: A teacher’s income is lower than a company employee’s and is at the same level as a civil servant’s. After practice: A teacher’s income is not as high as I thought.
Social status	11	Typical	Before practice: The social status of teachers is high, and teaching is a great profession. After practice: a teacher’s social status is so-so and is not as glorious as I imagined.
**Identity factors**			
Mentors at field school	12	General	Mentors have the most influence, and they are conscientious, attentive, and give me lots of guidance.
Students at field school	6	Variant	The most impressive aspect for me is my students; I am so touched by their concern for me.
**Identity roles**			
Emotions	8	Typical	I want to be a teacher, and I think it is meaningful. Teaching is a low paying and laborious job, but my thoughts on becoming a teacher have not changed.
Beliefs in teaching career	12	General	After I learned more about the contents, the environment and the conditions of a teacher’s job, my belief in teaching became stronger, and I had no hesitation.

*N = 12. General = all 12 cases represented, Typical = 8–11 cases represented, Variant = 4–7 cases represented*.

#### Changes in Identity

The first domain, the changes in identity, showed the changes of pre-service teachers’ professional identity before and after the internship. Specifically, there were two categories in this domain as follows: intrinsic value identity and extrinsic value identity. There were five sub-categories in the two categories. Intrinsic value identity consisted of work contents and work characteristics, and extrinsic value identity consisted of work environment, income, and social status. The five sub-categories are representative: work content was general; work characteristic, income, and social status were typical, and work environment was variant.

All 12 participants had a cognitive change in work content before and after their internships. The original sentences of some participants are cited here, as follows: “As for work content, I had only a very basic idea about it before the internship. I thought that the teachers only taught and managed the class. Through the internship, I learned that teachers also have to work on tasks that the school assigns to them.” Some participants “took part in teaching research under the guidance of the mentor in the internship, which enabled them to experience what it feels like to be a research-oriented teacher,” and understood more about the work contents of a teaching job, which “impressed me very much.” In sum, the understanding and identity of the teaching profession rose from vague to explicit.

Eleven participants spoke about a re-acquaintance with the characteristics of the teaching profession. Before the internship, they generally felt that “teaching is boring and sort of routine,” “in 3 years, the job becomes a mechanical repetition,” and “especially when it comes to teaching a course, the whole teaching life has to be spent dealing with only a few textbooks.” Through the internship, they discovered that “teaching can be creative and not just a simple repetition” or that there is “truly a lot to learn”; some participants even greatly improved their understanding of the professional requirements and attainment through the internship. They realized that “the more you learn, the more you can give to students; moreover, the broader the field you study, the higher you stand, and the deeper knowledge you have, and these can benefit the students.”

Of the 12 participants, 9 talked about income and the welfare of teachers. Before the internship, most participants thought, “a teacher’s income is at an average level in society, which is close to that of civil servants” and that “the welfare of a teacher is not as good as a company employee.” After the internship, participants’ understanding of the situation did not change much. Some participants even noted that “their understanding was more realistic than before,” that “as a profession, teaching is not so good and stable as I had imagined,” or that “a teacher’s hard work is not reasonably proportional to their financial reward.”

Eleven participants mentioned the social status of teachers. Before the internship, the participants thought that “teachers have a high social status and are respected by the society,” “the social status of teachers is high; teaching is a great profession.” However, after the internship, they thought that “a teacher’s social status is common, and teaching is just a common job; the teaching profession in fact is not as glorious as I thought.”

Only four participants mentioned their understanding of teachers’ work environment. They thought that “the work environment is good, and the social circle is unique – simple and pure, not complicated.” After the internship, these views remained the same.

#### The Factors of Identity

In the second domain under the effect domain—the factors of identity—one general category (mentor at the field school) and one variant category (students at the field school) were extracted.

As for the impact of mentor support on professional identity, all participants emphasized the important roles mentors and other teachers played in the field school. The original interview discourses are cited here as follows: Some participants argued that “responsible teaching attitude and comprehensive guidance from the mentor” were major factors to make him/her focus more on the teaching profession. Some participants were deeply moved by the mentors. They realized that “the most positive effect was from my mentor. He designed the course based on his ideas and creativity, activated the students and created a learning atmosphere in the classroom to make students more motivated to learn.” “Before, I focused more on arranging the teaching content but with the guidance of my mentor, I realized it was more important to communicate with the students in class and to make students more interested in learning.” One participant who completed his internship at a key middle school in Beijing talked about the deep thinking and high professional identity brought to him by the teachers at the field school: “the strongest effect from my mentor was his professionalism, sense of responsibility and attitude. The teaching profession depends on teachers’ conscience; you need to do your job from the heart, perform your full duty, not just one day or two, but forever.” Vice versa, two out of the twelve participants thought that “job burnout, lack of responsibilities and de-motivation of teachers at the field school” were the major factors impacted on his/her teaching initiatives. This finding confirmed the opposite side of the importance of a mentor.

Additionally, the current study also analyzed the importance of students at field school. Six out of the 12 participants spoke about how the students in their classes had deeply influenced them. Some participants said, “I communicated with the bad students in my class and found they communicated sincerely, and they were the ones I have the most contact with now.” A participant who completed the internship in a middle school in Hebei province felt that “the most impressive experience for me was the students; I remember once after P.E. class, I wanted a chair to sit on, but there was only one dirty chair in the classroom. A student took off his shirt to wipe it clean for me. I was so touched by that.” Many things like this happened during the internship. These things made participants realize the students’ care and understanding, which was the most important factor for participants to appreciate the teaching profession.

#### The Roles of Identity

The third domain, the roles of identity, showed the influence of internships on pre-service teachers’ future professional development. Specifically, there were two categories in this domain: emotional evaluation and belief in the teaching profession.

Of the 12 participants, 8 evaluated their emotions toward the teaching profession at the end of their internship. The emotions of the majority of participants changed from “good” or “neither like nor dislike” to “thinking highly of teachers,” “it is worthwhile to be a teacher,” and “we should respect and cherish the profession and do the job assuredly.” Some participants said that “I’d like to be a teacher; as a profession, it is meaningful,” “Teaching is a low paying and laborious job; sometimes there are complaints, but they are just for the sake of complaining. They do not affect my feelings for the job,” and “Compared with other professions, I prefer teaching.”

During the interviews, all 12 participants stated their beliefs in the teaching profession at the end of their internship. Most participants noted that the internship strengthened their beliefs in having a teaching profession. Some participants said that “the internship was a turning point for me; it made up my mind to continue trying to be an excellent teacher.” Other participants described their changes before and after the internship: “At the beginning, my family thought I was suited for a teaching job, and I did not reject that idea. After the internship, when I started to understand the ramifications, environment, and condition of a teaching job, my thinking on teaching in school became stronger and has not changed since then.” Other participants said that “my confidence was strengthened, and I learned a lot.” The internship showed that their beliefs in having a teaching career were strengthened.

## Discussion

The present study used a mixed qualitative and quantitative approach. The quantitative research investigated the changes of pre-service teachers’ professional identity before and after the internship. The qualitative research illustrated and elaborated the changes of professional identity in more detail; further analyzed the factors of professional identity and their roles in pre-service teachers’ professional development in the future; and revealed the relationships among pre-service teachers’ professional identity, mentor support, and professional commitment.

### Forming a Positive Yet Tense Professional Identity after Experiencing the Internship

The results of quantitative data showed that compared with professional identity before the internship, pre-service teachers’ professional identity increased after the internship; specifically, intrinsic value identity increased significantly, and extrinsic value identity kept steady, which was consistent with the results of qualitative data. The results of qualitative research indicated that pre-service teachers had a new understanding of the contents and characteristics of teaching work. These new features, communication with students, creativity of teaching, etc., had become attractive to pre-service teachers. These factors contributed to changing pre-service teachers’ stereotype of the teaching profession before experiencing the internship. Conversely, the harsh reality of the profession, such as income level, social status, and work environment, did not quite match the “greatness,” “high status,” and “middle-class” images pre-service teachers held before their internship, which even led to some pre-service teachers’ negative cognition of the teaching profession; however, the changes almost kept steady before and after the internship.

There were both similarities and differences in results between the present study and the study of Hong (2010). The changes of professional identity and the two dimensions in the study meant that pre-service teachers’ attitudes became more realistic after experiencing the internship, which was consistent with the results of Hong (2010). Hong (2010) analyzed the differences in the emotion dimension of professional identity between pre-service teachers experiencing the internship and those not experiencing the internship. The results indicated that pre-service teachers who had not experienced the internship were obviously too optimistic and underestimated the effect of educational situation on emotion; pre-service teachers who had experienced the internship had less idealized concepts.

However, inconsistent with the results of Hong (2010), the results of her cross-sectional study showed that there were no significant differences in the value dimension of professional identity between pre-service teachers who had not experienced student teaching and pre-service teachers who had experienced student teaching. This inconsistency may be related to different approaches; the present study used longitudinal design and excluded generational differences effectively. It was conducted to detect real changes in pre-service teachers’ professional identity before and after the internship. Furthermore, the inconsistent results also related to different samples, different measuring tools, and different internship modes.

As stated in the review, researchers tended to view professional value as a whole to measure previous studies on professional identity; however, the current study investigated pre-service teachers’ professional identity from intrinsic value identity and extrinsic value identity. Intrinsic value identity is a value judgment of a profession’s work attributes (e.g., work content, work pattern), whereas extrinsic value identity is a value judgment of a profession’s social attributes (e.g., work environment, income, and social status). The study showed that the two dimensions had different variation trend before and after the internship, which contributed to deepen understandings of pre-service teachers’ professional identity and changes of different dimensions.

In the current study, the cognition and evaluation of work contents and features reflected the intrinsic value of the teaching profession, whereas the cognition and evaluation of income, social status, and work environment reflected the extrinsic value of the teaching profession. Intrinsic value identity was strengthened whereas extrinsic value identity was kept steady relatively, even somewhat weakened in a certain sub-category throughout the internship. This result indicated that the interior of professional identity had a conflict change. The overall professional identity was positive and increasing, but it was likely to go through more inner tension after experiencing internship. Festinger (1957) proposed the approach-avoidance conflict characteristics of volitional behavior according to cognitive dissonance theory. This inner tension of professional identity could bring more uncertainty to the pre-service teachers’ career choices and professional commitment in the future. Pre-service teachers might improve their extrinsic value identity to help them integrate into the teaching profession completely or they might reduce their intrinsic value identity and then leave the teaching profession.

Additionally, the results also meant that it is important to enhance the design and plan of the internship. During field teaching practice, if pre-service teachers are provided multiple tasks and contents, they will have a chance to experience fully and deeply the kinds of characteristics of the teaching job that will have great significance for the development of professional identity, especially for cultivating and promoting their intrinsic value identity. Certainly, multiple and abundant tasks in the internship are closely related to the supports of the important others (e.g., mentors); otherwise, pre-service teachers will be frustrated, which will not be beneficial for the improvement of professional identity.

### Mentor Support at Field School Effectively Facilitates Professional Identity

The results of qualitative data indicated that teaching guidance and work attitudes of the mentor at the field schools played a critical role in pre-service teachers’ professional identity. Many participants realized that “the most positive effect was from the ideas and creativity of their mentors” and that “they were conscientious and did their job from their heart,” which impressed with participants. Some participants cited the influence of students of school and experienced “being moved” and “accomplishment.” The results indicated that the support of mentors at the field schools was important in the internship stage.

The results were supported by some studies. Izadinia (2016) conducted a study by interviewing seven pre-service teachers and mentors. The results showed that the mentoring relationship was an important influencing factor in pre-service teachers’ professional identity. Specifically, when the mentoring relationship was more positive, the pre-service teachers felt more confident as teachers and their professional identities were higher, whereas, their professional identity and confidence both declined when the mentoring relationship was negative. Stufflebeam (2000) emphasized the importance of mentor support and proposed that these supports also influenced teaching efficacy, professional orientation, and professional commitment, which was proved by Schepens et al. (2009). The results showed that mentor support played an important role in pre-service teachers’ professional efficacy, professional commitment, and professional orientation. The three variables were the components of professional identity. An empirical study indicated that the communication and feedback of the mentor at the field school had an important effect on pre-service teachers’ affective commitment; especially, mentors’ communication and feedback were far more important for pre-service teachers than campus teaching (Christophersen et al., 2016). Furthermore, students at the field school were also considered to be one of the most motivating factors influencing teachers’ professional identity and professional development (Proweller and Mitchener, 2004). The interactions of new teachers with students deeply influenced their teaching perspective, self-confidence, and work satisfaction (Bullough, 2001).

In conclusion, this result revealed that the supports of the mentor were important. These supports would not only directly influence pre-service teachers’ professional identity but also could have a profound effect on their future professional commitment. The sources of supports are not limited to the mentors but also include the entire teacher community, such as school leaders, teaching assistants, students, and parents of students (Avalos and Aylwin, 2007). Williams (2010) emphasized that there were abundant social practices and social relationships in field teaching practice, which was important not only to form professional identities but also to make a successful career transition. Providing supports for pre-service teachers, including providing guidance in the initial teaching jobs for novice teachers, adapting school culture, communicating the class plan to expert teachers, etc., decreased the difficulty of transition from student to teacher (Johnson and Ridley, 2004). These supportive strategies should be adopted by every school.

### Professional Commitment of Pre-service Teachers Strengthened by Internship

The results of the qualitative data indicated that pre-service teachers’ emotional evaluation was more positive and that they had a firmer belief in their teaching career after the internship. According to previous definitions of professional commitment in studies (e.g., Meyer et al., 1993; Van Huizen, 2000), professional commitment refers to the extent of one’s individual emotional connection with one’s profession and the extent of one’s unwillingness to change professions. This study’s findings showed that pre-service teachers’ professional commitment was strengthened.

Professional commitment has a direct and important effect on individual professional decision in the future. Rots et al. (2007) found that commitment to teaching, especially the initial commitment to teaching pre-service teachers obtained after completing learning and training during teacher education, was closely related to whether one chose to be a teacher in the future. The initial commitment to teaching was an important predictor of teacher leaving his job in the earlier stage of professional development (Rots et al., 2010). Professional commitment directly influenced an individual’s career decision-making, and decision to stay in or leave a job, meanwhile, also was influenced by many factors, such as teachers’ supports (e.g., supports of educator, supports of mentor) and professional efficacy. Professional commitment would play an important role between these factors and career decision-making. For example, results from primary and middle school teachers in Netherlands indicated that affective commitment mediated the relationships between classroom self-efficacy and responsibility to remain in teaching, between change in level of motivation and responsibility to remain, between relationship satisfaction and responsibility to remain in a structural model (Canrinus et al., 2012).

Based on previous studies, the qualitative study further revealed the potential relationships among pre-service teachers’ professional commitment, professional identity, and mentor support. That is, mentor supports predicted pre-service teachers’ professional identity and commitment, and professional identity mediated the relationship between mentor support and professional commitment. Certainly, the influencing mechanism was based on the case interview, and the result will need to be tested by a quantitative study in the future. Meanwhile, the results provided evidence for distinguishing between professional identity and professional commitment. Professional identity referred to the fact that individuals made judgments or evaluations on different characteristics of profession, while professional commitment mainly focused on professional affection, including aspiration of remaining in the current job and the degree of enjoyment of the job (Blau, 1985). The two concepts focused on different psychological components, were different in the emergence and formation period, and embodied different developmental stage of professional attitude. Accordingly, from the perspective of professional attitude, the results in the current study also implied the development and transition of pre-service teachers’ professional attitude after the internship, which provided inspiration for exploring studies about professional attitude transition in the future.

### Conclusion and Implications for Teacher Education

The current study used mixed methods to explore the changes in pre-service teachers’ professional identity, and used qualitative study to explore the factors of professional identity, and the roles of professional identity in professional development and commitment during the internship. The results indicated that compared with professional identity before the internship, pre-service teachers’ professional identity increased after the internship; specifically, intrinsic value identity increased while extrinsic value identity remained steady. Mentor supports in field school were important factors. Regarding the roles of professional identity, pre-service teachers’ professional commitment, including affective evaluation of teaching profession and teaching belief both increased.

The results of the present study have several important implications for promoting teacher education programs, and especially for improving the effectiveness of field teaching practice. First, teacher-training institutions should further expand the contents of field practice to provide pre-service teachers with more opportunities to participate in various kinds of practical work and expand pre-service teachers’ understanding of the teaching profession. Second, the school should arrange proficient mentors for pre-service teachers, excellent teachers’ supports should contribute to promote pre-service teachers’ professional identity and commitment, and the sources of the supports should not be limited to teacher groups. The ways of support should be diversified. Third, as an essential part of teacher education, internship should be expanded throughout all years in college. This is particularly important in teacher education in China. The internship is assigned in the last year of teacher education and continues for 3 months. Therefore, the short time and lateness for participating in an internship limit pre-service teacher’s understanding of the teaching profession. Additionally, the extrinsic values of the teaching profession need to be further improved. These extrinsic factors have no significant roles in pre-service teachers’ professional identity during the internship, but the inner tension of professional identity cannot be neglected. The government and educational administration should formulate relevant educational policies and further improve the extrinsic values of the teaching profession.

### Limitations and Future Directions

There are several limitations regarding this study that must be noted. The first limitation is the loss of the sample in the longitudinal study. Given the limited sample conditions, the present study extended sampling range and differences in demographic variables as far as possible to improve the representativeness of the sample. Meanwhile, from the point of sampling bias, the loss of participants in the longitudinal research was random. However, the number of participants (*n* = 98) was still low for a quantitative study, which could have led to unsteady results.

More care should be taken to avoid losing the sample in the future. Second, the number and representativeness of the sample in the qualitative research need to be improved. Hill et al. (2005) argued that researchers should randomly select participants from a homogeneous total group. The participants should have rich knowledge of the research topics and recent relevant experience. In other words, the sampling should be based on a set standard. The current study met Hill’s criterion, and the number of participants also met the smallest number some researchers have deemed acceptable (Kuzel, 1992; Morse, 1994). Additionally, the present study used mixed methods to investigate the changes in pre-service teachers’ professional identity. However, the relationships among professional identity, mentor support, and professional commitment during the internship still need to be tested using quantitative data in the future.

## Author Contributions

HZ designed the study, analyzed the data, wrote and revised the article. XZ designed the study, collected and analyzed the data, interpretated the data, wrote the article. All authors approved of the publishment of the article and ensured that the questions related to the accuracy or integrity of any part of the work were appropriately investigated and resolved.

## Conflict of Interest Statement

The authors declare that the research was conducted in the absence of any commercial or financial relationships that could be construed as a potential conflict of interest.
